# Supplementary material for: Mechanism of self/nonself-discrimination in Brassica self-incompatibility
Source: Nat Commun. 2020 Oct 1;11:4916. doi: 10.1038/s41467-020-18698-w (PMC7530648; doi:10.1038/s41467-020-18698-w)
Supplement: Supplementary file 1 — Supplementary Information [file 41467_2020_18698_MOESM1_ESM.pdf]

Supplementary Materials

Mechanism of self/nonself-discrimination in *Brassica* self-incompatibility

Murase *et al.*

Supplementary Figures 1–11
Supplementary Table 1
Supplementary References

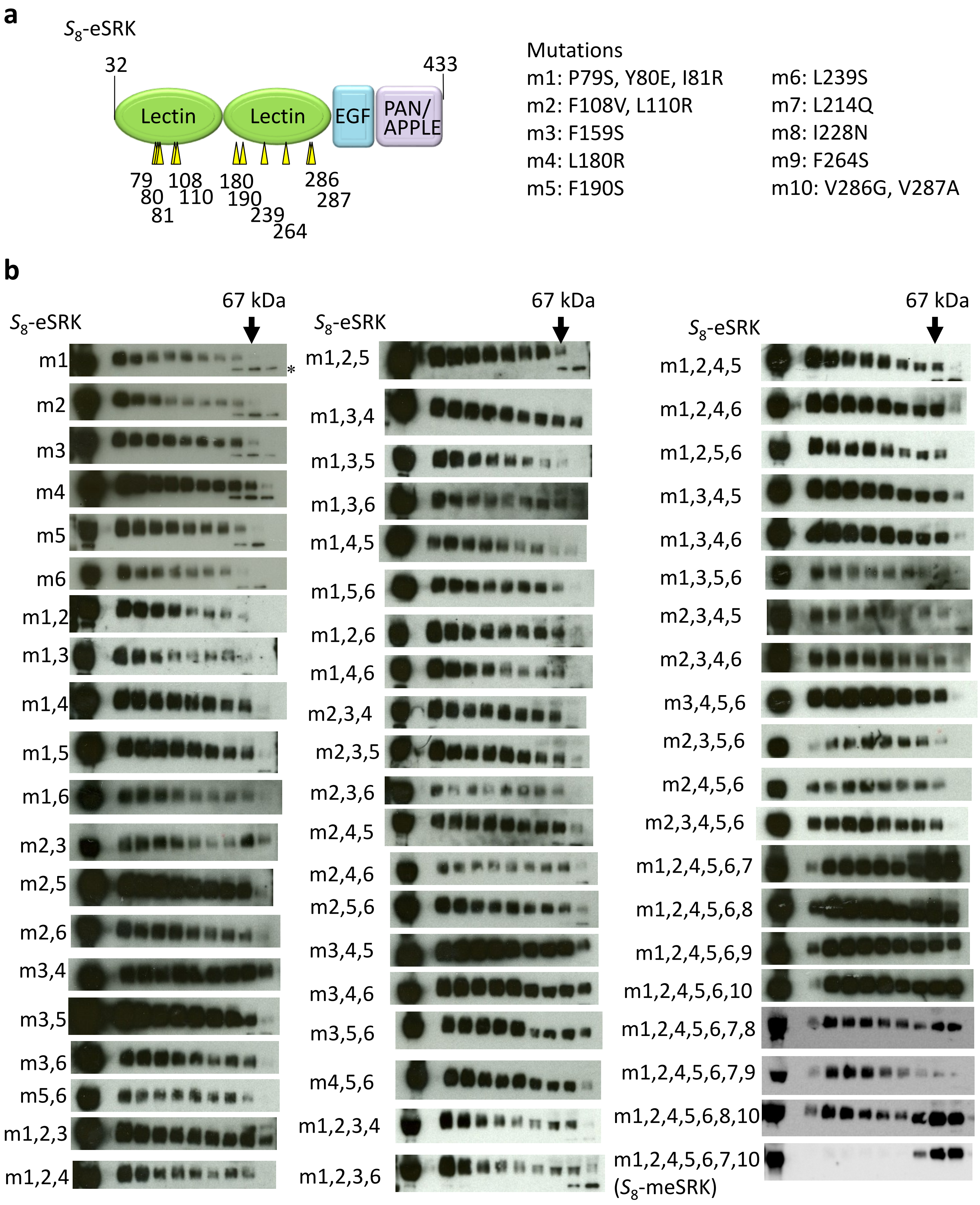

Supplementary Fig. 1

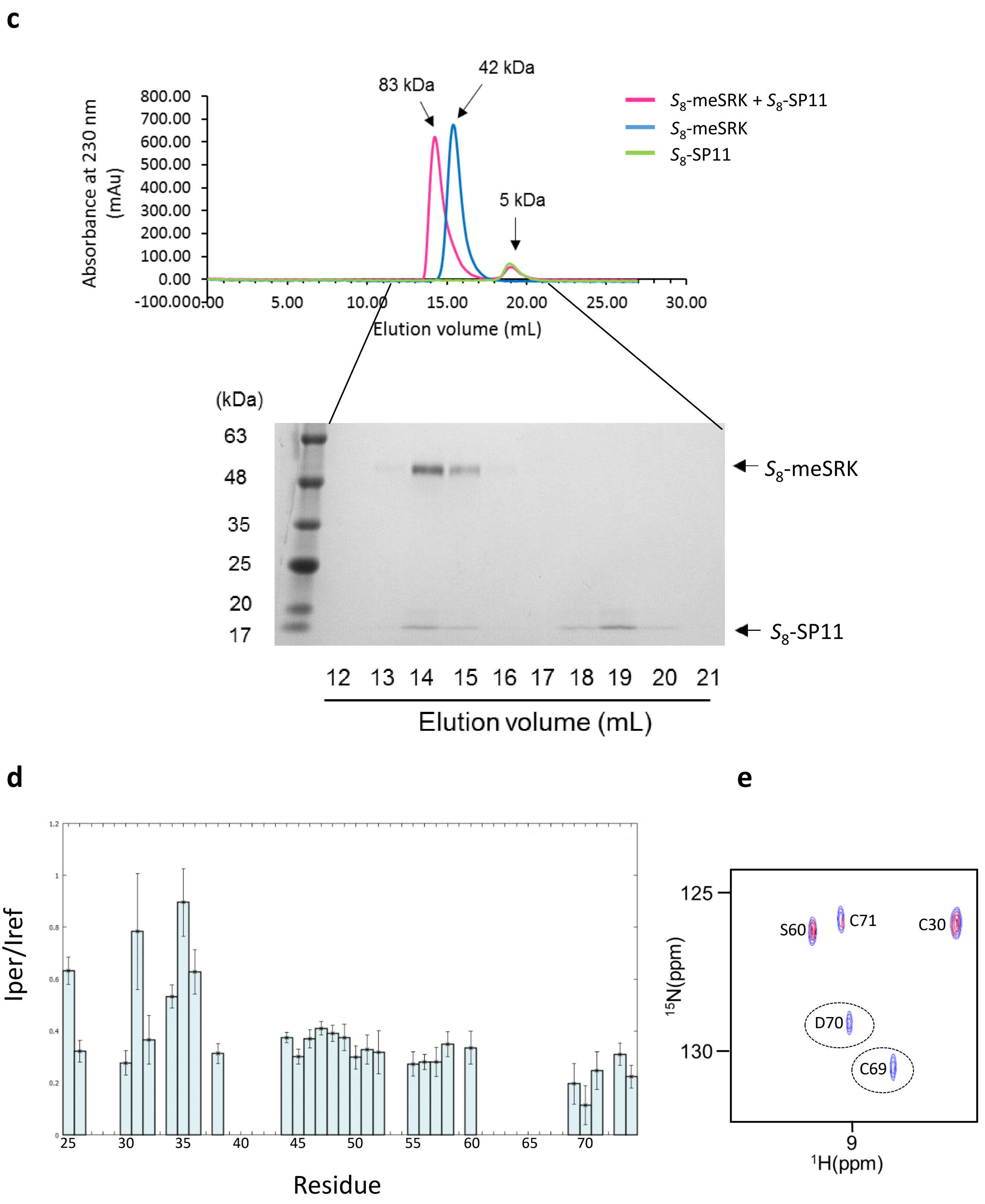

Supplementary Fig. 1

Supplementary Fig. 1 *S_8*-meSRK expression and recognition of *S_8*-SP11. a, Left, schematic diagram of S_8-eSRK constructs. Arrowheads show the positions of mutations used in S_8-meSRK. Right, list of mutations used for screening of S_8-eSRK expression shown in b. **b**, Screening of S_8-eSRK constructs for stable overexpression in Sf9 cells. Numbers in the construct names indicate that the constructs have the corresponding mutations listed in a. Asterisk shows non-specific bands. **c**, Gel-filtration analysis of S_8-meSRK, S_8-SP11, and S_8-meSRK–S_8-SP11 proteins using a calibrated Superdex 200 column. Upper panel shows the merged chromatogram of the samples. Arrows show the calculated molecular size of each peak. Lower panel shows a Coomassie blue–stained SDS-PAGE gel of the separated fractions from chromatography of S_8-meSRK–S_8-SP11 proteins. **d**, Chemical shift perturbation analysis of S_8-SP11. The signal intensities of the ^{1}H-^{15}N HSQC spectrum of S_8-SP11 were reduced upon addition of unlabeled S_8-meSRK. Signal reduction versus amino acid residue is shown for S_8-SP11. The data are represented by the intensity ratio Iper/Iref; Iper and Iref were measured in the presence and absence of S_8-meSRK, respectively. The error bars were calculated based on the signal-to-noise ratios. **e**, Selected region of overlay of 2D ^{1}H–^{15}N HSQC spectra of the ^{15}N-labeled S_8-SP11 in the presence (red) and absence (blue) of S_8-meSRK. The representative drastically perturbed (i.e., reduced) signals are marked by dotted circles. The S_8-SP11 and S_8-meSRK concentrations were 70 μM and 35 μM, respectively. The spectra were recorded at 900 MHz at the ^{1}H frequency.

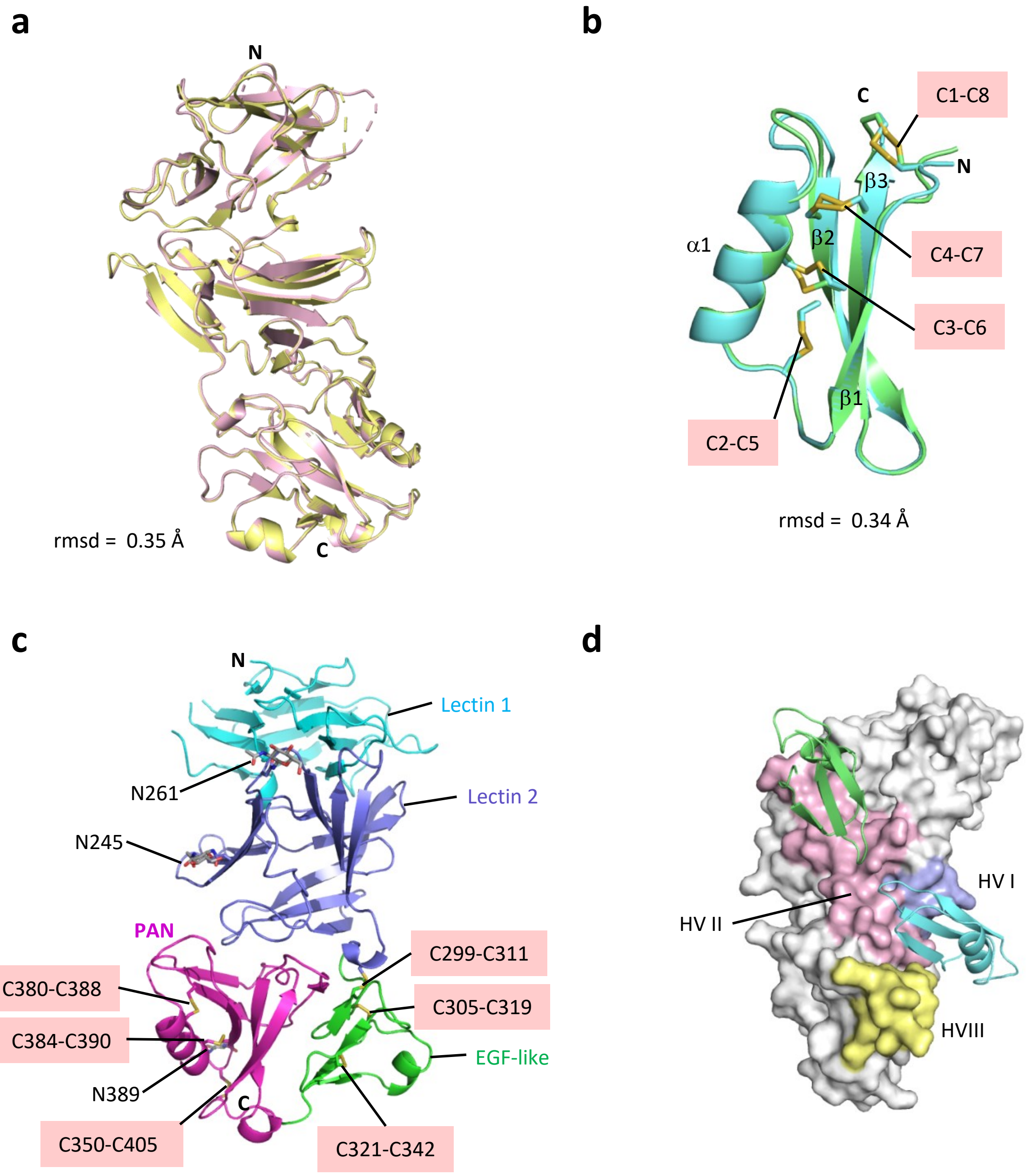

Supplementary Fig. 2 Structural features of S_8-meSRK–S_8-SP11 complex. a, Superimposition of the two S_8-meSRK molecules in the heterotetramer of S_8-meSRK–S_8-SP11, colored in pink and yellow. Dashed lines show disordered regions. **b**, Superimposition of two S_8-SP11 molecules in the heterotetramer of S_8-meSRK–S_8-SP11, colored in cyan and green. Eight cysteine side chains forming disulfide bonds are represented by stick models. **c**, Domain organization of S_8-meSRK. Lectin 1, Lectin 2, EGF-like, and PAN domains are colored in cyan, purple, green, and magenta, respectively. Cysteines forming disulfide bonds and sugar chains are represented as stick models. **d**, HV regions forms an SP11 binding surface on S_8-meSRK. HV regions are shown in purple (HV I), salmon pink (HV II), and yellow (HV III). S_8-SP11 molecules are colored in cyan and green.

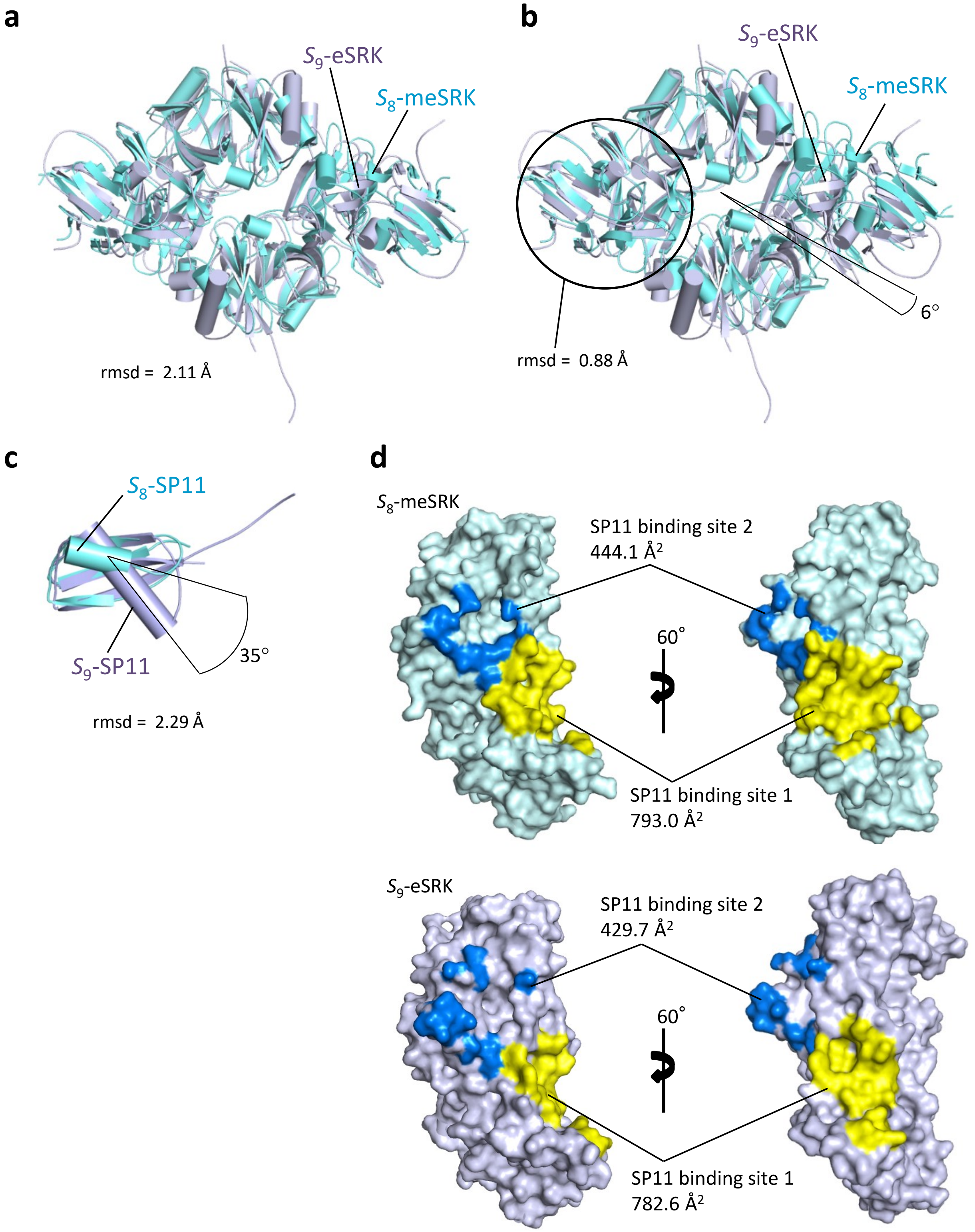

Supplementary Fig. 3

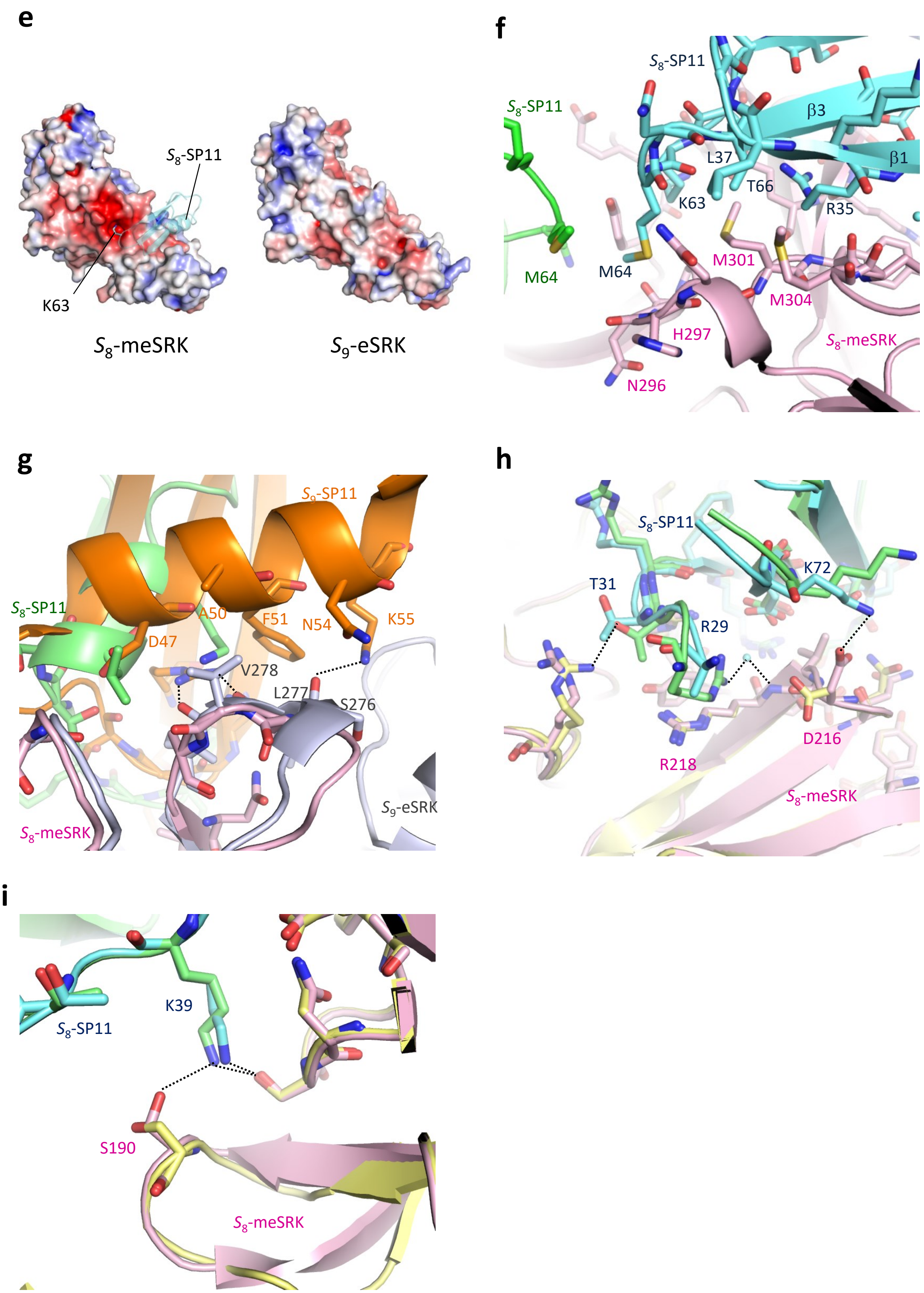

Supplementary Fig. 3

j

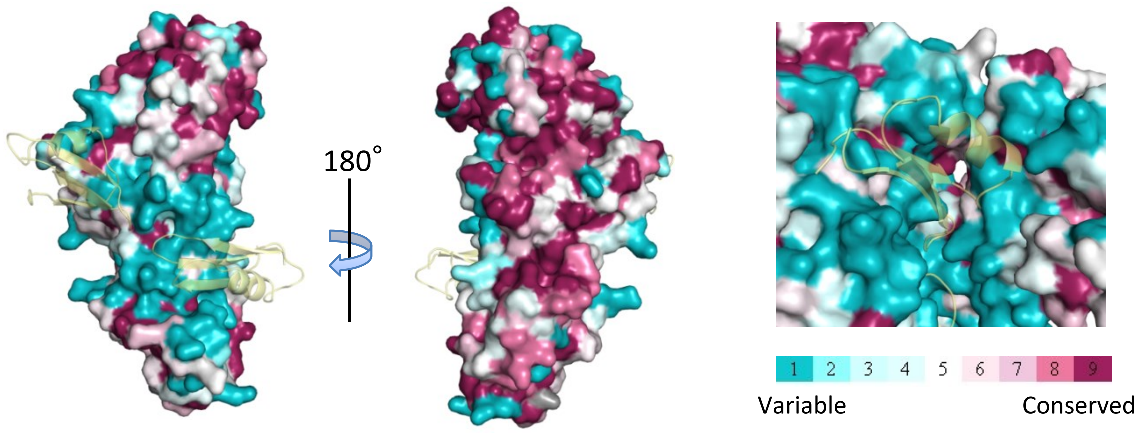

Supplementary Fig. 3 Comparison of S_8-meSRK–S_8-SP11 and S_9-eSRK–S_9-SP11 complexes. a, **b**, S_8-meSRK–S_8-SP11 (cyan) and S_9-eSRK–S_9-SP11 (purple) complexes are superimposed using C_α atoms of whole complexes (a) and single eSRK molecules (b). eSRK molecules used for superimposition are enclosed by a circle. **c**, S_8-SP11 (cyan) is superimposed on S_9-SP11 (purple). **d**, Molecular surfaces of S_8-meSRK (cyan) and S_9-eSRK (purple). SP11 binding sites 1 and 2 are colored in yellow and blue, respectively. **e**, Electrostatic potential surfaces of S_8-meSRK (left) and S_9-eSRK (right). S_8-SP11 is shown in cyan. **f**, Close-up view of the center of the S_8-meSRK–S_8-SP11 complex. S_8-meSRK is shown in pink, and S_8-SP11 molecules in cyan and green. **g**, Close-up view of HV III regions of S_8-meSRK (pink) and S_9-eSRK (silver). S_8-SP11 and S_9-SP11 are shown in green and orange, respectively. **h**, **i**, Differences in ligand–receptor interaction between the two heterodimers in the S_8-meSRK–S_8-SP11 complex. The heterodimers are superimposed based on the C_α atoms of each S_8-meSRK. S_8-meSRK–S_8-SP11 pairs are shown in pink–cyan and yellow–green, respectively. The side chain of Arg29 in S_8-SP11, shown in cyan, is disordered. g–i, Dotted lines represent hydrogen bonds. Water molecules are shown in small cyan spheres. **j**, Conservation profile of *Brassica* eSRK proteins. Conservation scores, calculated with the ConSurf program using 30 *B. rapa* SRK sequences, are shown in color on the molecular surface of S_8-meSRK. S_8-SP11 molecules are shown in yellow.

a

```
                    20                  40
S8-SRK     1 --MQGVRYIYHHFYTSLLVFVVMILFRSALSIYINTLSSTESLTISNNRTLVSPGDVFEL
S9-SRK     1 --MKGVRNIYHHSYTFLLVFFVMILFRPAFSLST--LSSTESLTISSNRTLVSPGNIFEL
S12-SRK    1 MKCVRNIYYQSYTFSFVLAFVVLILFHPAISMHFNTLLSTESLTISGNRTLVSPGHVFEL
S52-SRK    1 -------------TSFLLVYVVMILFHPALAIYINTLSATESLTISSNRTLVSPGNVFEL
S60-SRK    1 MKGVHNIYHHSYTFSFLLVFLALILFHPALSTYVNTMSSSESLTISSNRTLVSPGGVFEL

             60                  80                  100
S8-SRK    59 GFFTPGSSSRWYLGIWYKKLPYITYVWVANRDNPLSNSTGTLKISGNNLFLLGDSNKSIW
S9-SRK    57 GFFRTN--SRWYLGMWYKKLSGRTYVWVANRDNPLSNSIGTLKISNMNLVLLDHSNKSVW
S12-SRK   61 GFFKNTLNSRWYLGIWYKNLSDRTYVWVANRDSSLSNAIGTLKFSGSNLVLRGRSNKFVW
S52-SRK   48 GFFITNSSSRWYLGIWYKKLTDRTYVWVANRDNPLSSSTGTLKISGNNLVIFGHSNKSVW
S60-SRK   61 GFFKPSGRSRWYLGIWYKKVSQKTYAWVANRDNPLSNSIGTLKISGNNLVLLGQSNNTVW

             120                 140                 160
S8-SRK   119 STNLTRGNERSPVVAELLANGNFVMRDSNNNDASGFLWQSFDYPTDTLLPEMKLGYDLKT
S9-SRK   115 STNLTRENVRSPVVAELLANGNFVVRDP-----SGFLWQSFDYPTDTLLPEMKLGYDLKT
S12-SRK  121 STNLTRGNERSPVVAELLANGNFVIRYSYNNDASGFLWQSFDFPTDTLLPEMKLGYYLKT
S52-SRK  108 STNLTIGNERSPVVAELLANGNFVMRDPNNNEASGFLWQSFDYPTDTLLPEMKLGYDLKT
S60-SRK  121 STNLTRENVRSPVIAELLPNGNFVMRYSNNKDSSGFLWQSFDFPTDTLLPEMKLGYDFKT

             180                 200                 220
S8-SRK   179 GLNRFLTSSRNFDDPSSGDYSYKLEPRR-LPEFYLLL----GDVREHRSGPWNGIQFSGI
S9-SRK   170 GLNRFLVSWRSSDDPSSGDFSYKLDIQRGLPEFYTFK----DNTLVHRTGPWNGIRFSGI
S12-SRK  181 GLNRFLTSWRNFDDPSSGEFSYKLETRR-LPEFYLLK----NGSPGQRSGPWNGVQFSGI
S52-SRK  168 GLNRFLISWRSSDDPSSGEITYKLEPRR-FPEFYIFS----DDFRVHRIGPWNGIGFIGI
S60-SRK  181 GRNRFLTSWRSYDDPSSGKFTYELDIQTGLPEFILINRFLNQRVVMQRSGPWNGIEFSGI
                                             HV I

             240                 260                 280
S8-SRK   234 PEDQKLSYMVYNFTENSEEVAYTFRMTNNSFYSRLTINSEGYLERLTWAPSSVVWNVFWS
S9-SRK   226 PEEQQLSYMVYNFTENSEEVAYTFLVTNNSIYSRLTINFSGFFERLTWTPSLVIWNPIWS
S12-SRK  236 PEDQTLSYMVYNFTENSEEVAYTFRMTDNSIYSRIQLSPEGLLERLTWTPTSGTWNLFWS
S52-SRK  223 PEDQNSTYIVYNFTENSEEVAYSFRMTNNSIYSRLIITSEGYFQRLMWTPSTEIWQVFWS
S60-SRK  241 PEVQGLNYMVYNYTENSEEIAYSFQMTNQSIYSRLTVS-DYTLNRFTRIPPSWGWSLFWS
                                                            HV II

             300                 320                 340
S8-SRK   294 SPN-HQCDMYRMCGPYSYCDVNTSPSCNCIQGFNPGNVQQWALRNQISGCKRRTRLSCNG
S9-SRK   286 SPASFQCDPYMICGPGSYCDVNTLPLCNCIQGFKPLNVQEWDMRDHTRGCIRRTRLSCRG
S12-SRK  296 APVDIQCDVYMTCGPYAYCDVNTSPVCNCIQGFMPFDMQQWALRDGTGGCIRRTRLSCSS
S52-SRK  283 SPMSLQCDPYRICGPYAYCDESTSPMCICIQGFDPKNRQQWDLRSHASGCIRRTRLRCSG
S60-SRK  300 LP-TDVCDSLYFCGSYSYCDLNTSPYCNCIRGFVPKNRQRWDLRDGSHGCVRTTQMSCSG
                                                   HV III

             360                 380                 400
S8-SRK   353 DGFTRMKNIKLPDTRMAIVDRSIGLKECEKRCLSDCNCTAFANADIRNRVTGCVIWTGEL
S9-SRK   346 DGFTRMKNMKLPETTMATVDRSIGVKECEKKCLSDCNCTAFANADIRDGGTGCVIWTGRL
S12-SRK  356 DGFTRMKNMKLPDTKMAIVDRSIDVKECEKRCLSDCNCTAFANADIRNGGTGCVTWTGEL
S52-SRK  343 DGFTRMKNMKLPDTTTAIVDRSIGVKECEKRCLSDCNCTAFANADIRNGGTGCVIWTGEL
S60-SRK  359 DGFLRLNNMNLPDTKTASVDRTIDVKKCEEKCLSDCNCTSFATADVRNGGLGCVFWTGDL

             420                 440
S8-SRK   413 EDMRNYAEGGQDLYVRLAAADLVKKRNGNWKIISLI
S9-SRK   406 DDMRNYAVSGQDLYVRLAAADVVEKRTANGKIVSLI
S12-SRK  416 EDIRNYIGNGQDLYVRLAAADLVKKRKANGKIISLI
S52-SRK  403 EDIRTYVADGQDLYVRLAAADLVRKRNANGKIVSLI
S60-SRK  419 VEIRKQAVVGQDLYVRLNAADLDFSSGEKRDRTGTI
```

Supplementary Fig. 4

b

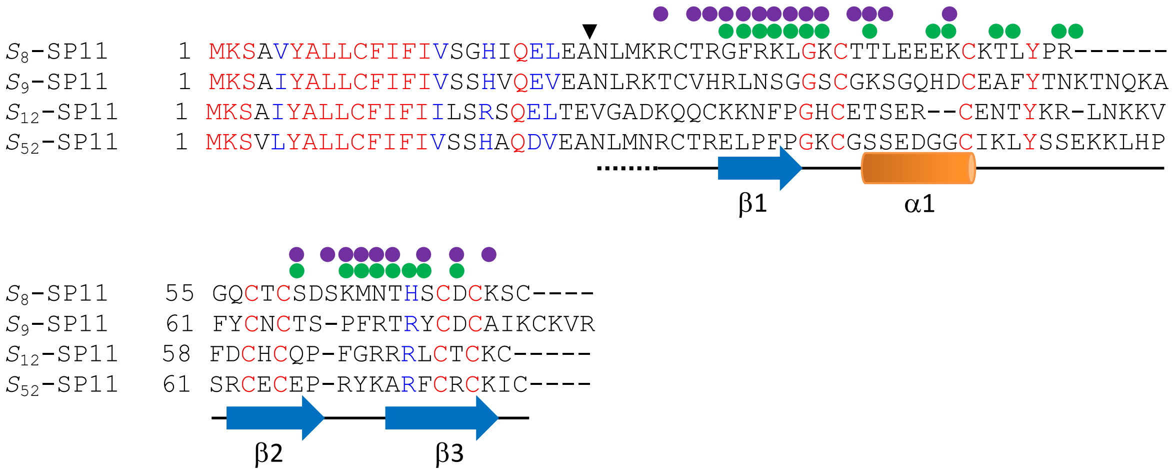

Supplementary Fig. 4 Sequence alignments of *Brassica* SRK and SP11. a, Sequence alignment of *B. rapa* SRK ectodomains. Contact amino acids against the cognate SP11 are shown as magenta (S_8) and cyan (S_9) circles. The black boxes indicate three HV regions. Cysteine residues forming disulfide bonds are connected by lines. Yellow arrowheads and upper numbers show the positions of S_8-SRK. The positions of the 11 amino acid mutations in S_8-meSRK are indicated by blue boxes. **b**, Sequence alignment of *B. rapa* SP11 proteins. Amino acids contacting with eSRK are shown by purple (S_8) and green (S_9) circles. Arrowhead shows the endpoint of the signal peptide. Arrows and cylinder indicate the β-strands and α-helix of S_8-SP11, respectively. Dotted line shows the disordered region in the crystal structure of the S_8-meSRK–S_8-SP11 complex.

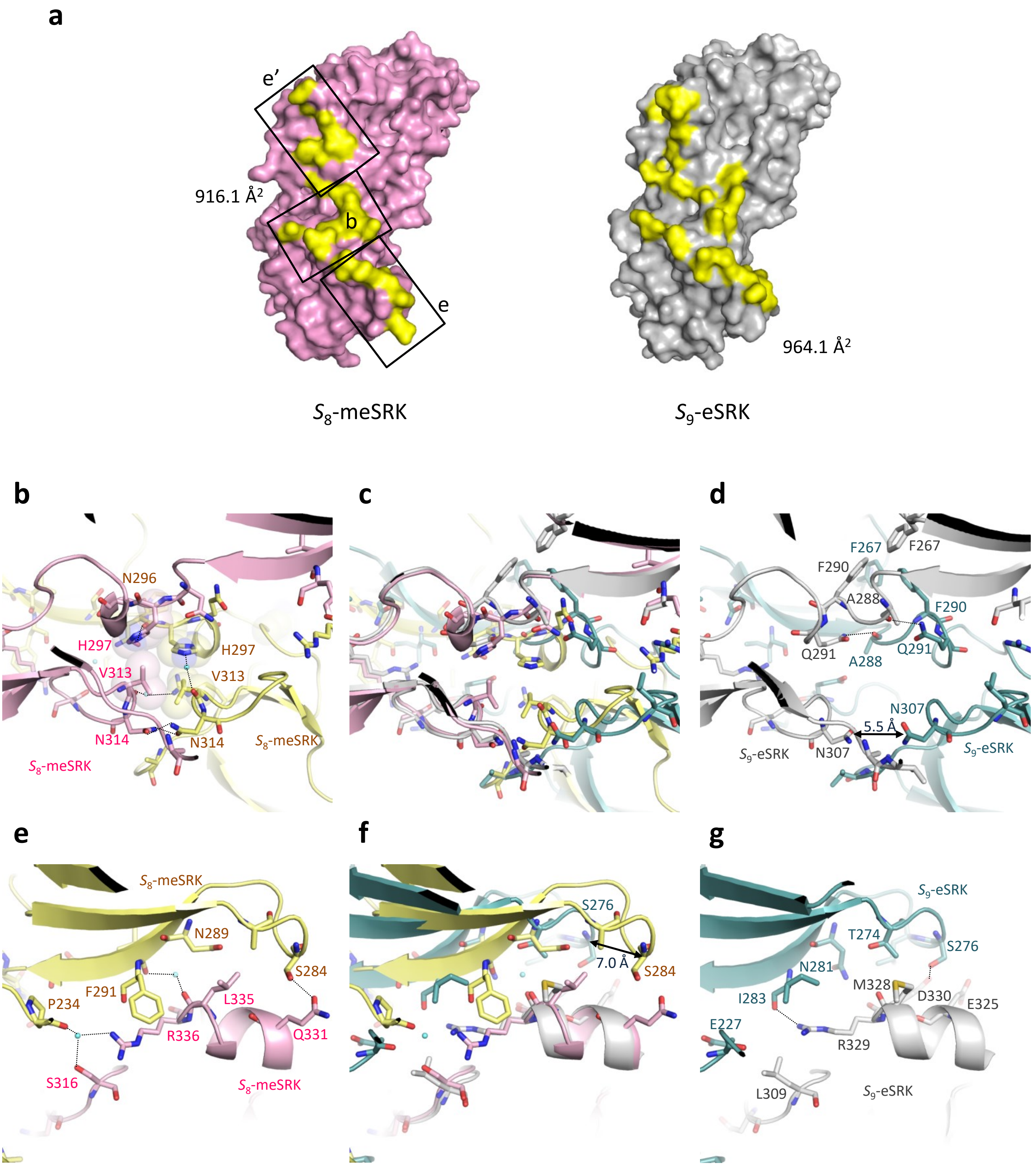

Supplementary Fig. 5

h

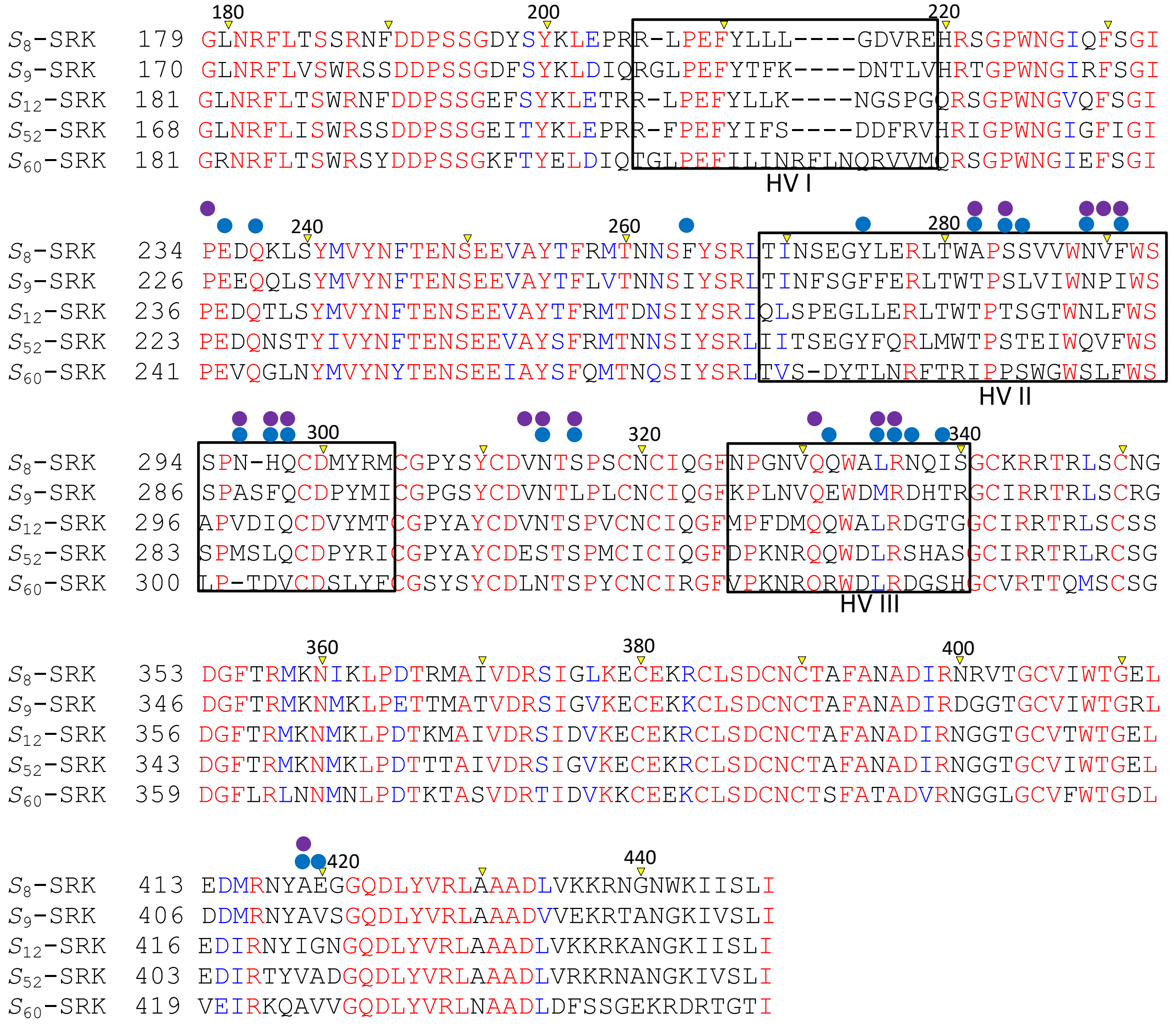

Supplementary Fig. 5 Comparison of homodimerization interfaces between *S*$_8$-meSRK and *S*$_9$-eSRK. a, Molecular surfaces of *S*$_8$-meSRK (left) and *S*$_9$-eSRK (right). Dimerization surfaces are colored in yellow. Details of the interactions enclosed by boxes are shown in b and e. Box e' indicates the symmetrical region of box e in the *S*$_8$-meSRK dimer. **b–g**, Close-up views of eSRK dimer interfaces. *S*$_8$-meSRK is shown in pink and yellow, and *S*$_9$-eSRK in silver and cyan. b, e, Homodimer interface of *S*$_8$-meSRK. c, f, Superimpositions of *S*$_8$-meSRK (pink) and *S*$_9$-eSRK (silver). d, g, Snapshot of *S*$_9$-eSRK homodimer interface, shown as in c and f. Dotted lines represent hydrogen bonds. Water molecules are shown as small cyan spheres. **h**, Sequence alignment of *B. rapa* SRK ectodomains. Amino acids involved in eSRK–eSRK interactions are shown in purple (*S*$_8$) and blue (*S*$_9$) circles. Three HV regions are shown in boxes. Yellow arrowheads and upper numbers show the positions of *S*$_8$-SRK.

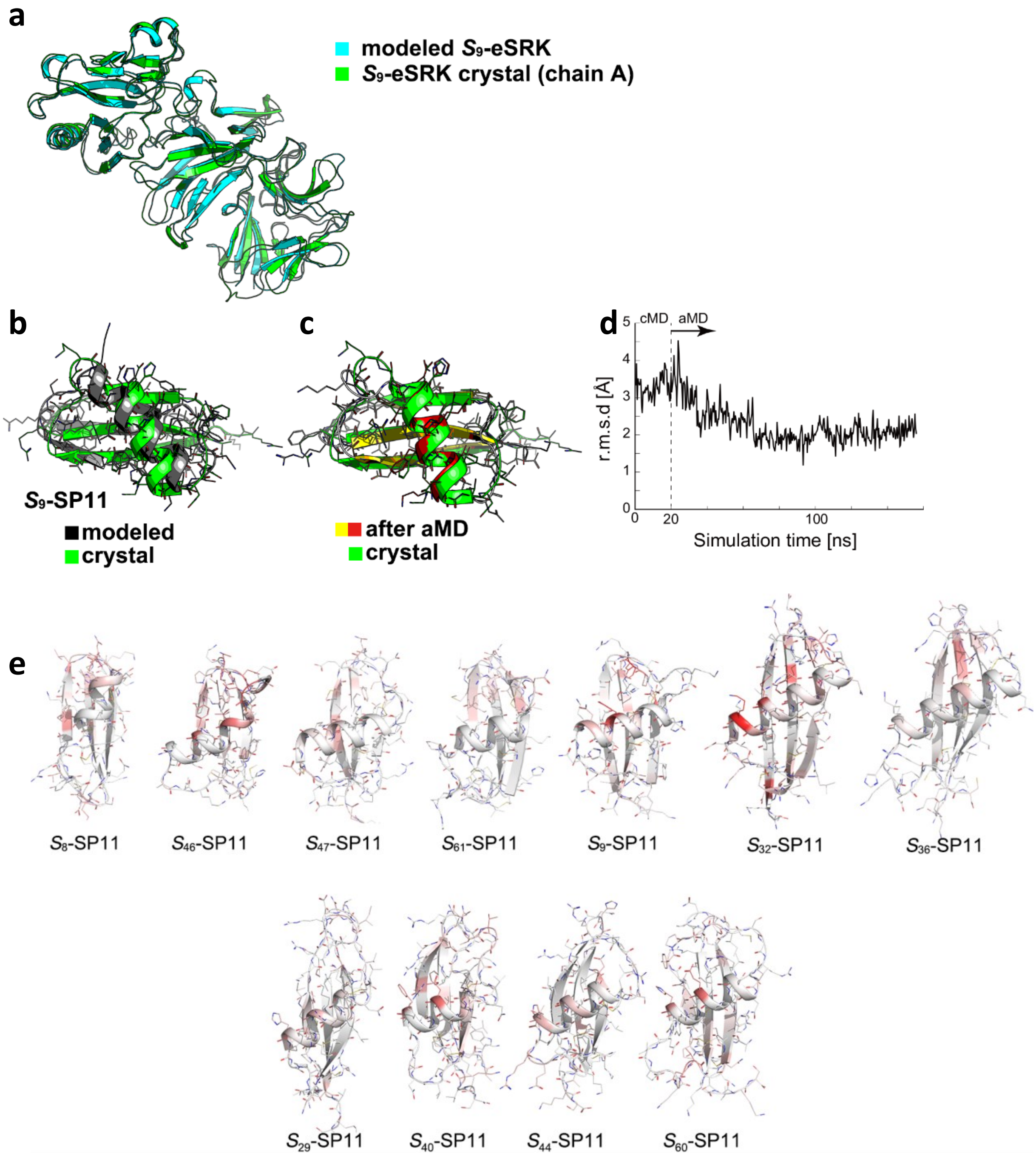

Supplementary Fig. 6 Modeling of various SP11 structures by aMD simulation. **a**, Structure of S_9-eSRK modeled using the crystal structure of S_8-meSRK (chain A). **b**, Initial structure of S_9-SP11, generated by homology modeling with S_8-SP11 as a template, is shown in black. Crystal structure of S_9-SP11 is depicted in green. **c**, Converged S_9-SP11 model obtained by aMD at 150 ns starting with the initial structure is shown in red (helix) and yellow (strand). **d**, Plots of rmsd over 150-ns aMD simulations. The aMD was conducted after 20-ns conventional MD simulations. The rmsd values were calculated for all Cα atoms of the crystal structure of S_9-SP11. **e**, Modeled SP11 structures (S_{32}, S_{36}, S_{46}, S_{47}, S_{61}, S_{29}, S_{40}, S_{44}, and S_{60}) after aMD improvement. Important residues for binding to the self-eSRK calculated by MM–GBSA are shown in red.

SRK

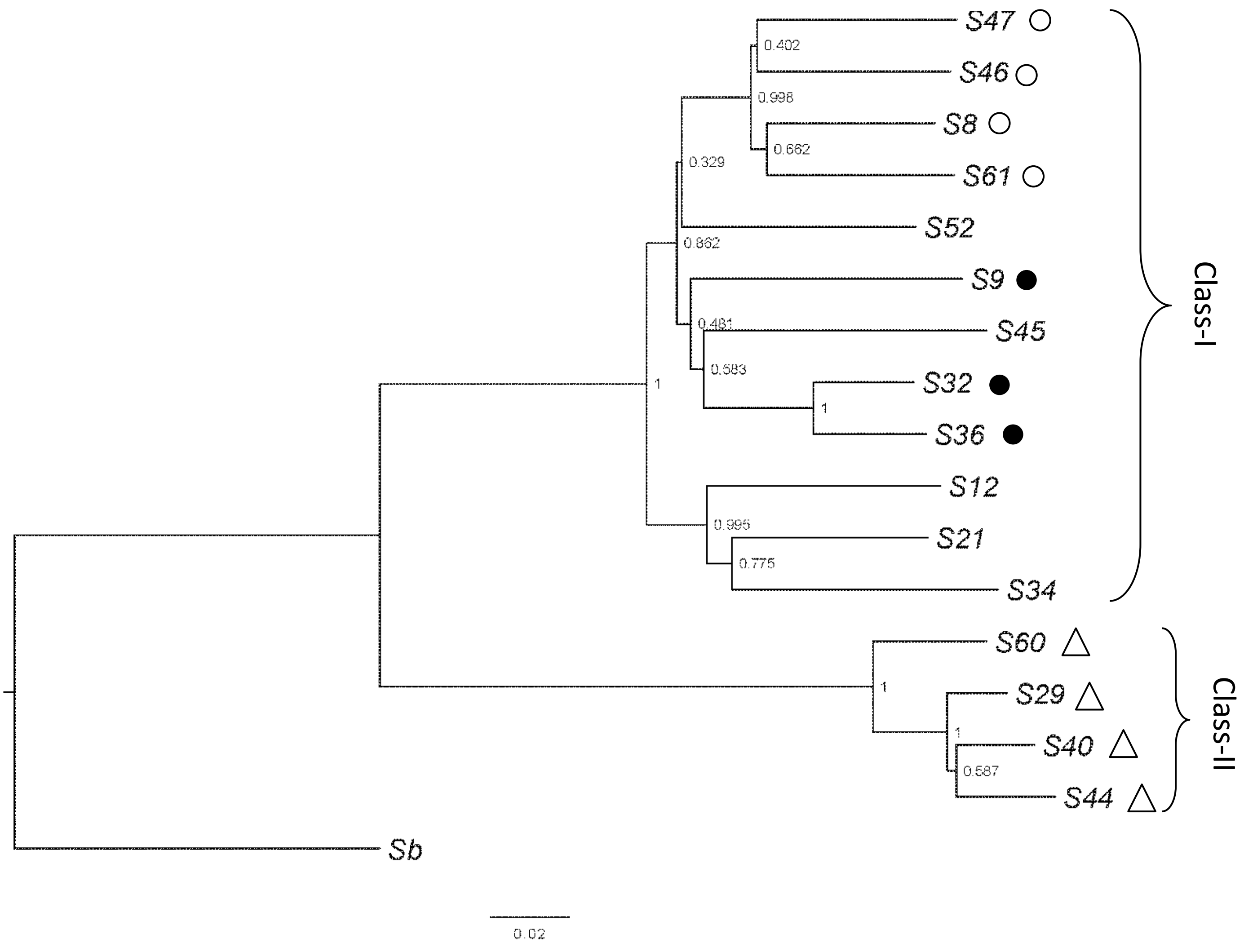

Supplementary Fig. 7

SP11

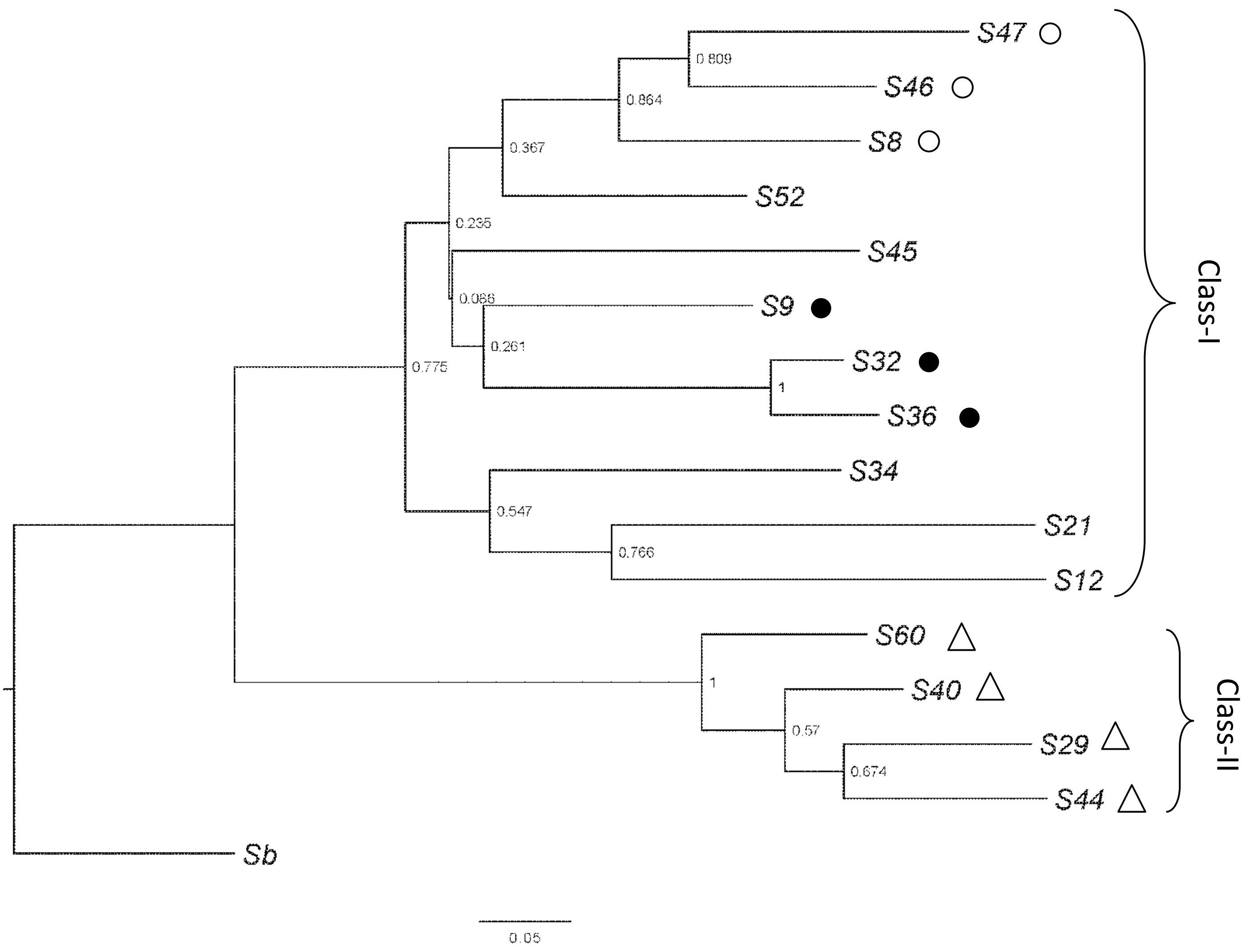

Supplementary Fig. 7 Phylogenetic trees of *Brassica SRK* and *SP11*. *S*-haplotypes categorized in the S_8-, S_9-, and class-II subgroups by MM–GBSA analysis are located in the same clades. White circles, black circles, and white triangles show the member of S_8-, S_9-, and class-II subgroups analyzed in this study, respectively. S_b-*SRK* and S_b-*SP11* sequences from *Arabidopsis lyrata* were used as the root. Sequences and accession numbers used in these trees are listed in Supplementary Data 1. S_{61}-*SP11* sequence was excluded from the tree because only a partial sequence was available.

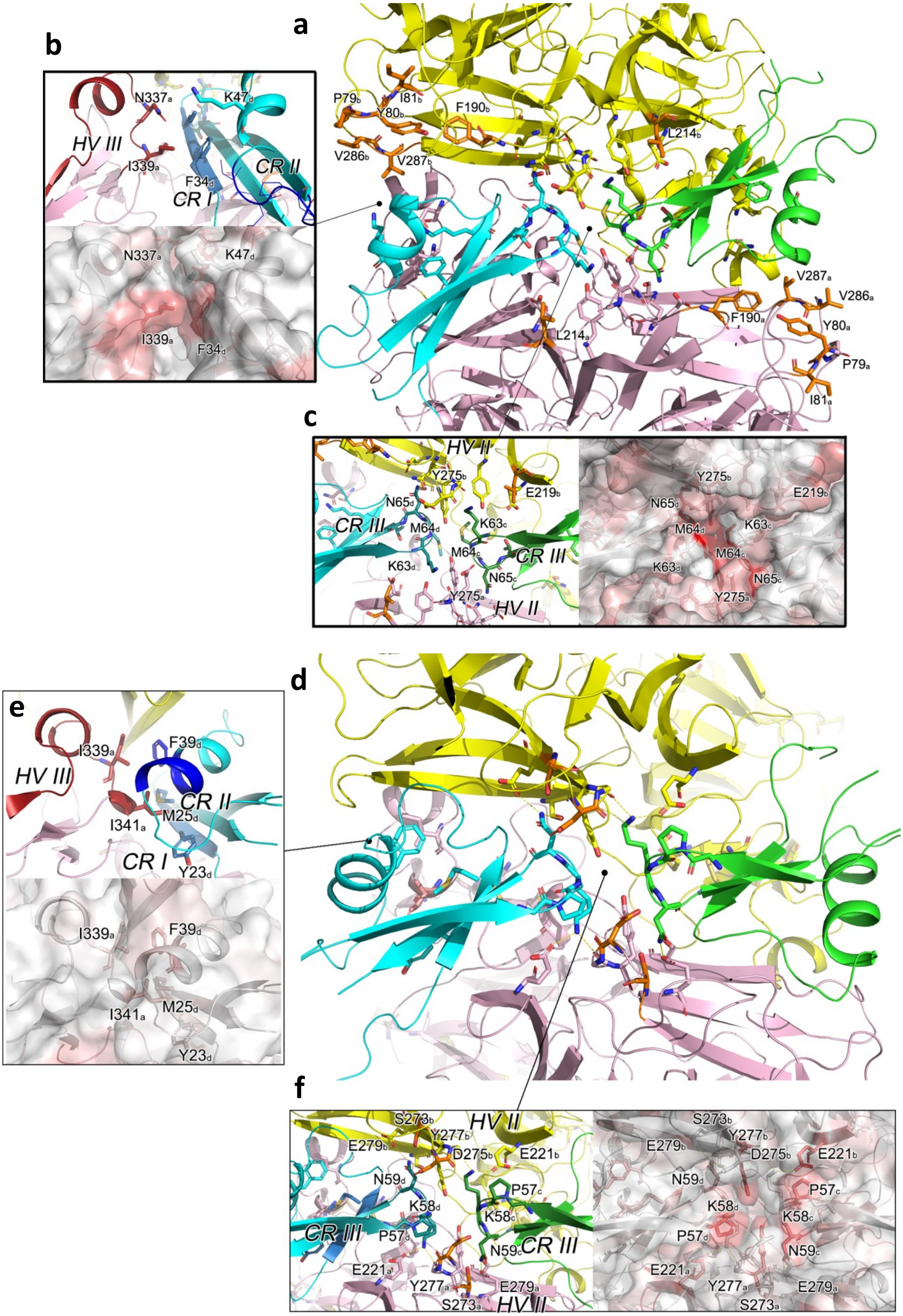

Supplementary Fig. 8

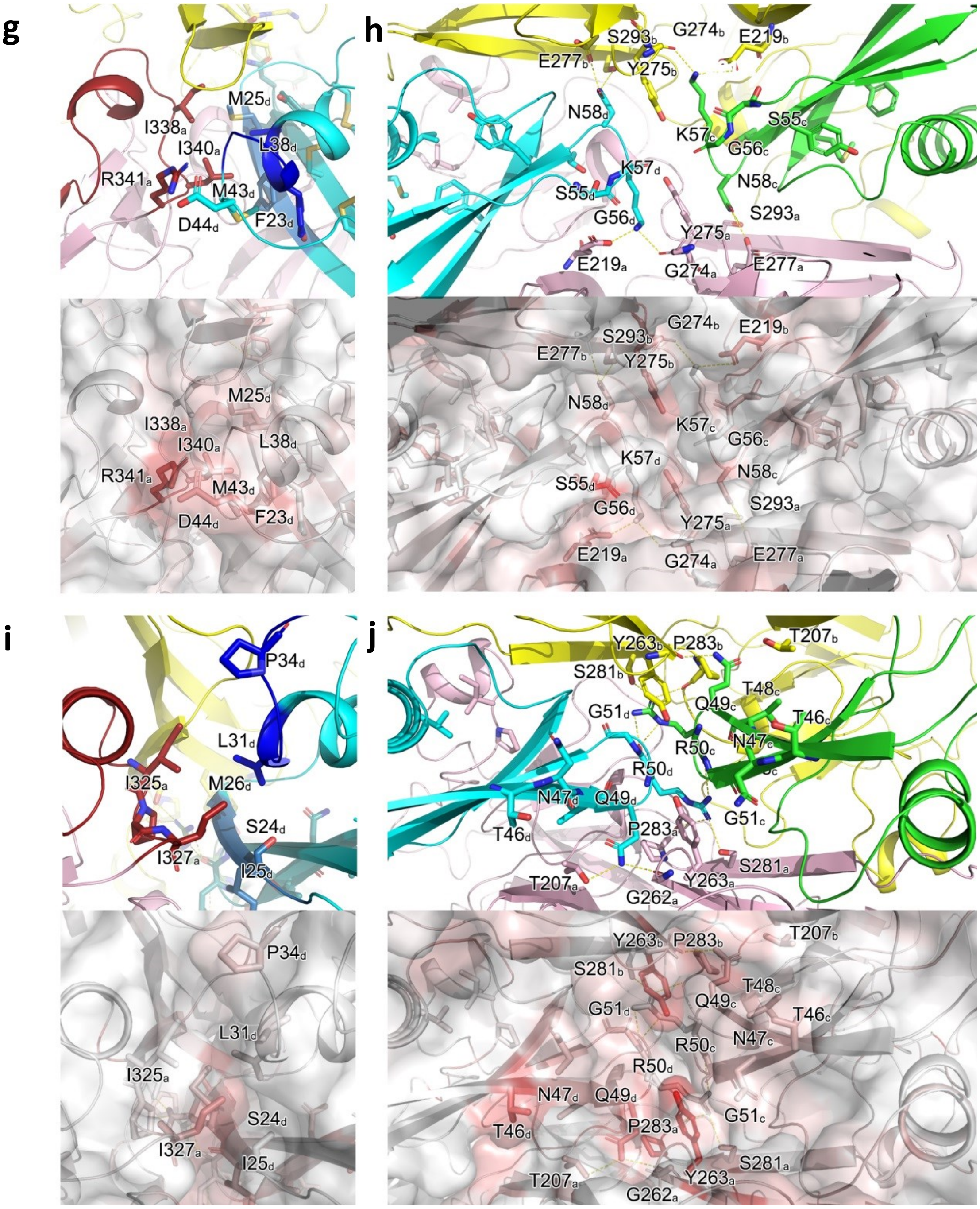

Supplementary Fig. 8 eSRK–SP11 model structures in S_8-subgroup. Modeled complex structures of S_8- (a–c), S_{46}- (d–f), S_{47}- (g, h), and S_{61}- (i, j) haplotypes are shown. Cyan and green represent SP11, and yellow and pink represent eSRK. **a**, **d**, Overall structure of S_8- (a) and S_{46}- (d) complex structures after 150-ns MD simulations. Mutated residues in S_8-meSRK (a) and in S_{46}-eSRK (d) used for pull-down assays shown in Fig. 4b are indicated in orange. **b**, **e**, **g**, **i**, Top panels show close-up views of the interfaces around eSRK-HV III, SP11-CR I, and SP11-CR II in S_8- (b), S_{46}- (e), S_{47}- (g), and S_{61}- (i) complexes, respectively. Ruby, marine, and bule represent HV III, CR I, and CR II, respectively. **c**, **f**, **h**, **j**, Left (c, f) and top (h, j) panels show close-up views of the interfaces where two eSRK and two SP11 molecules make contact in S_8- (c), S_{46}- (f), S_{47}- (h), and S_{61}- (j) complexes, respectively. Important residues for ΔG calculated by MM–GBSA are shown in red (c, f, right; h, j, bottom). Subscripts to the right of residue numbers indicate chain ID in the complex (a, b, eSRK; c, d, SP11).

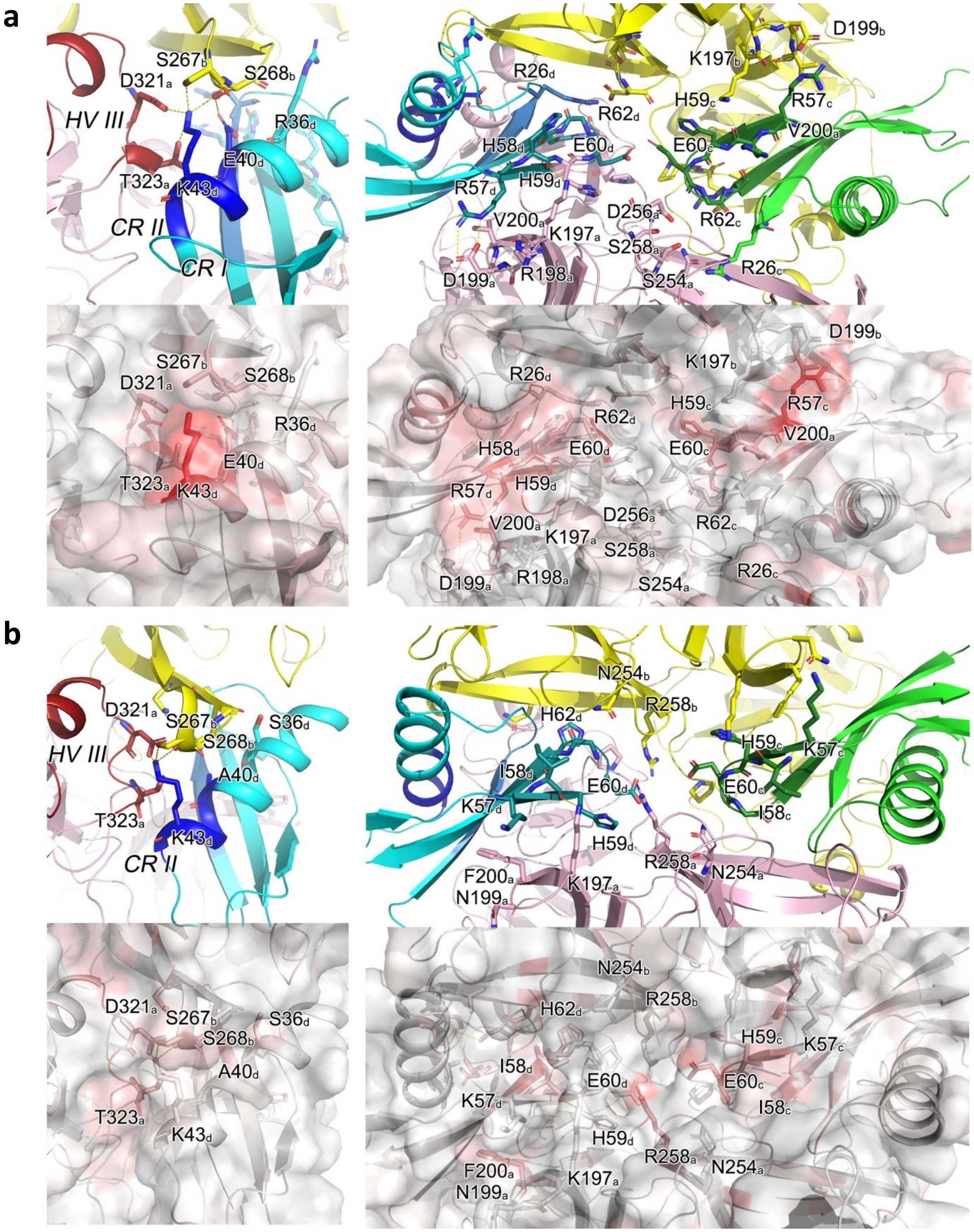

Supplementary Fig. 9 eSRK–SP11 complex structure models in S_9-subgroup. Model structures of S_{32}- (**a**) and S_{36}- (**b**) complexes. Cyan and green represent SP11, whereas yellow and pink represent eSRK. Close-up views of the interfaces around eSRK-HV III, SP11-CR I, and SP11-CR II are shown in the left upper panels, and close-up views of the interfaces where two eSRK and two SP11 molecules make contact are shown in the right upper panels. Dotted lines represent hydrogen bonds. Important residues for ΔG calculated by MM–GBSA are shown in red (bottom). Subscripts to the right of residue numbers indicate chain ID in the complex (a, b, eSRK; c, d, SP11).

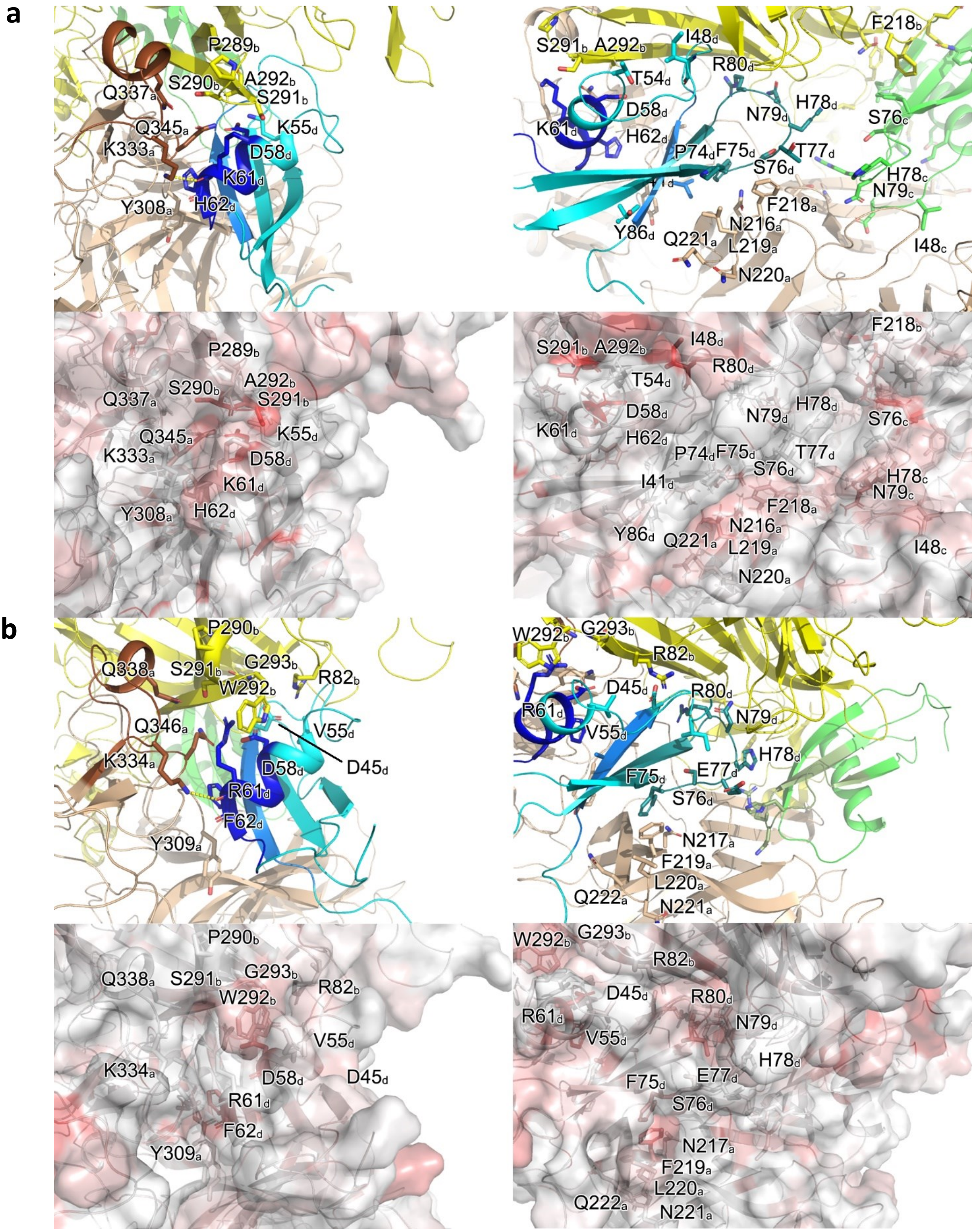

Supplementary Fig. 10

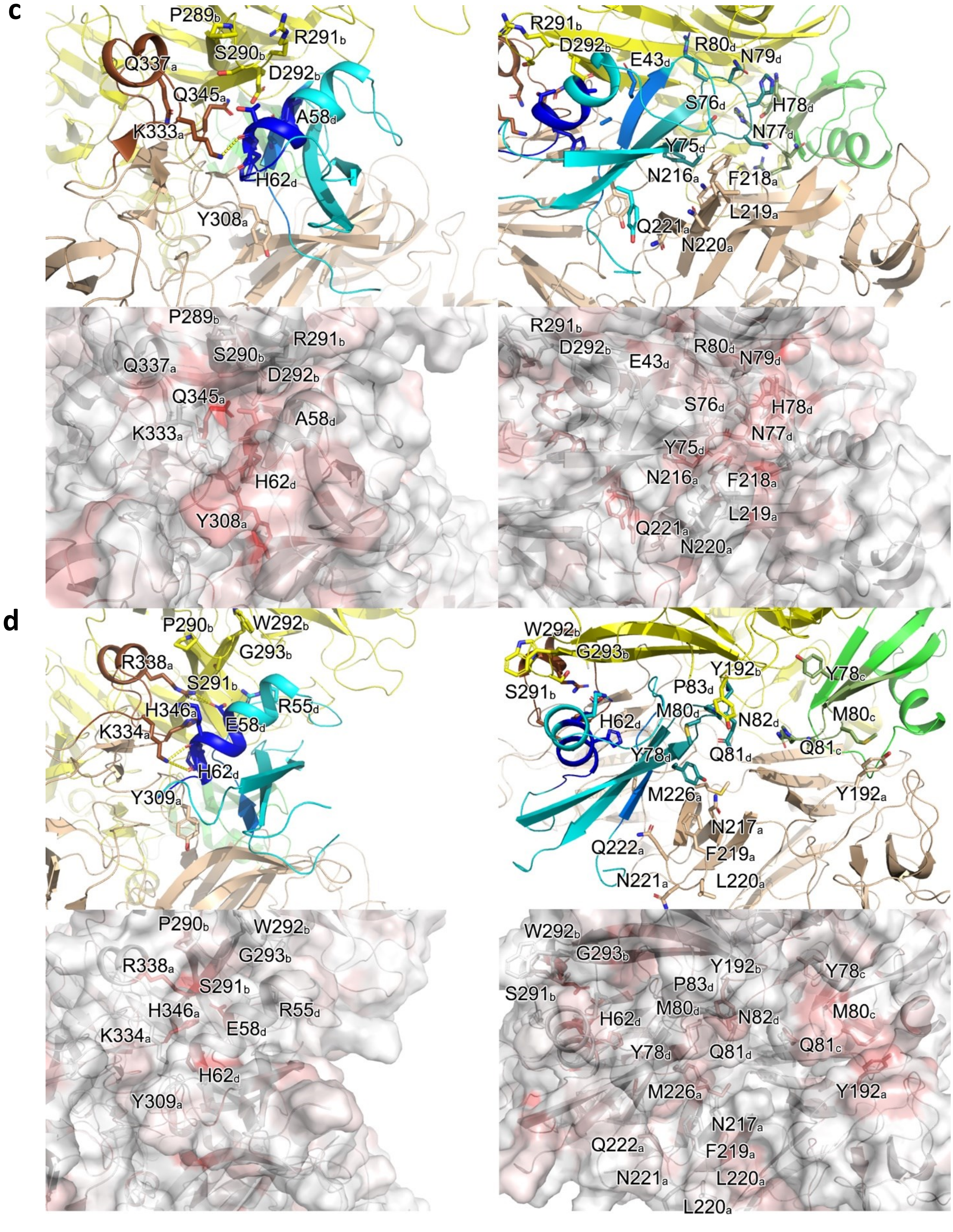

Supplementary Fig. 10

Supplementary Fig. 10 eSRK–SP11 complex structure models in the class-II subgroup. Model structures of S_{29}- (**a**), S_{40}- (**b**), S_{44}- (**c**), and S_{60}- (**d**) complexes. Cyan and green represent SP11, while yellow and light orange represent eSRK. Close-up views of the interfaces around eSRK-HV III, SP11-CR I, and SP11-CR II are shown in the left upper panels, while close-up views of the interfaces where two eSRK and two SP11 molecules make contact are shown in the right upper panels. Ruby, marine, and bule represent HV III, CR I, and CR II, respectively. Dotted lines represent hydrogen bonds. Important residues for ΔG calculated by MM–GBSA are shown in red (bottom). Subscripts to the right of residue numbers indicate chain ID in the complex (a, b, eSRK; c, d, SP11).

Fig. 1a

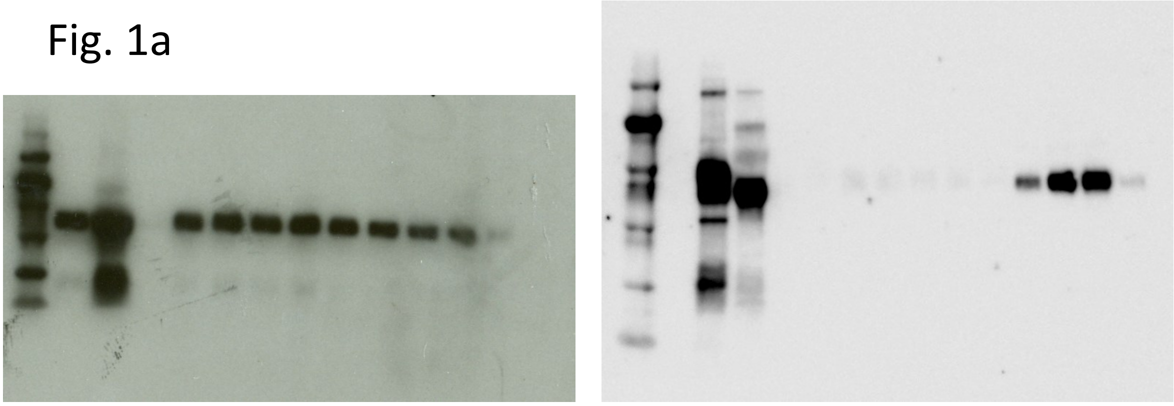

Fig. 1b

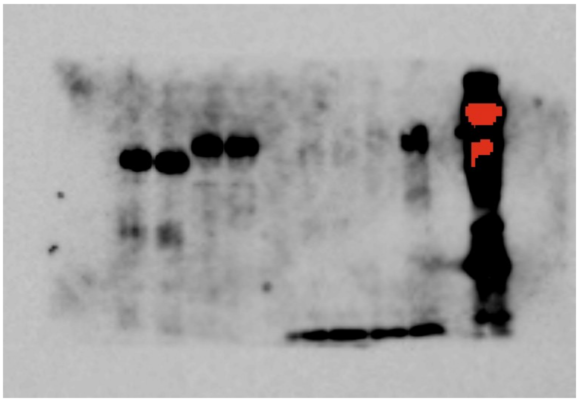

Fig. 2j

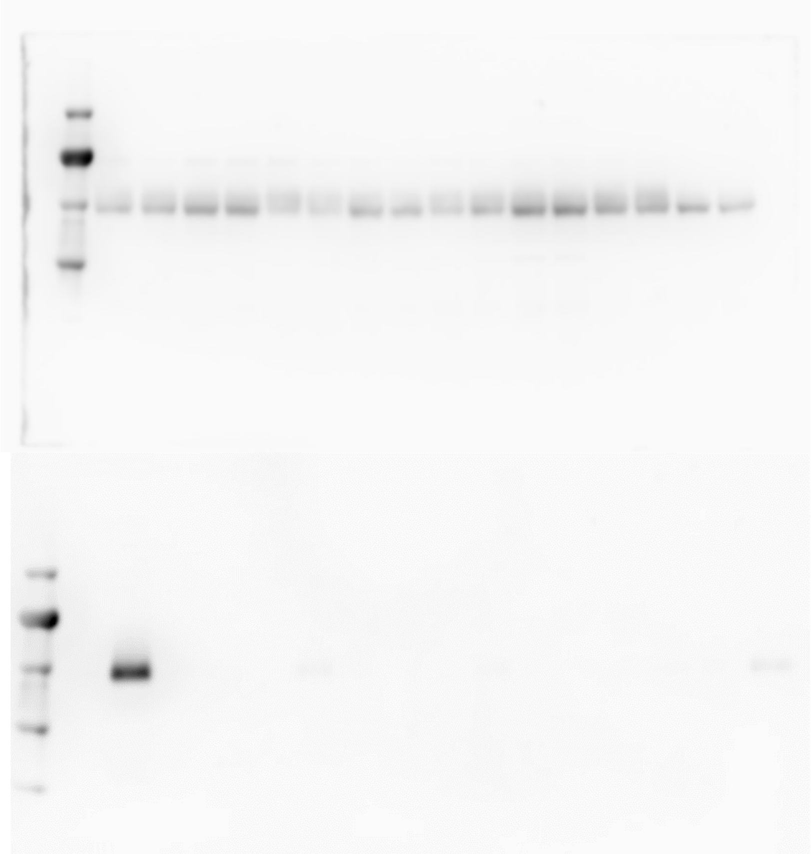

Supplementary Fig. 11

Fig. 4b

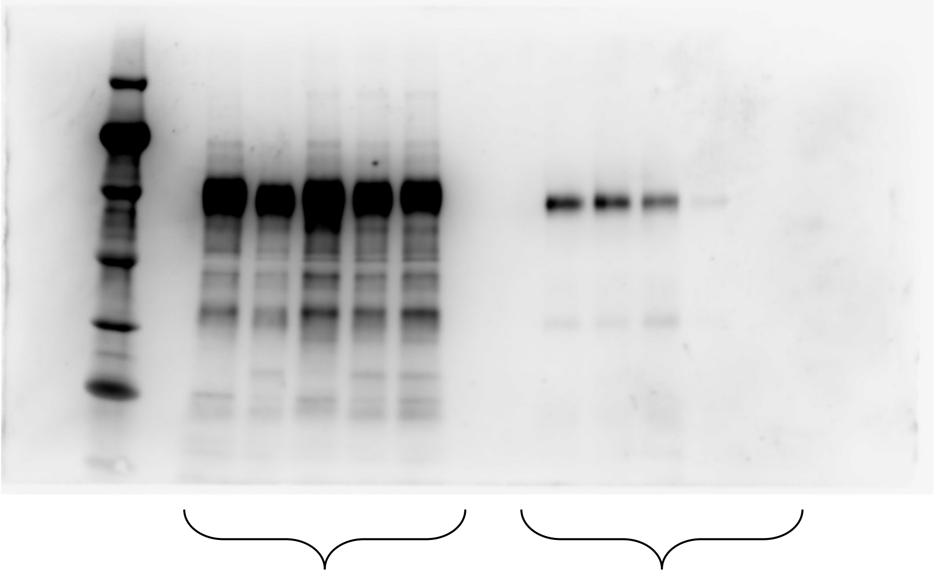

Upper panel Lower panel

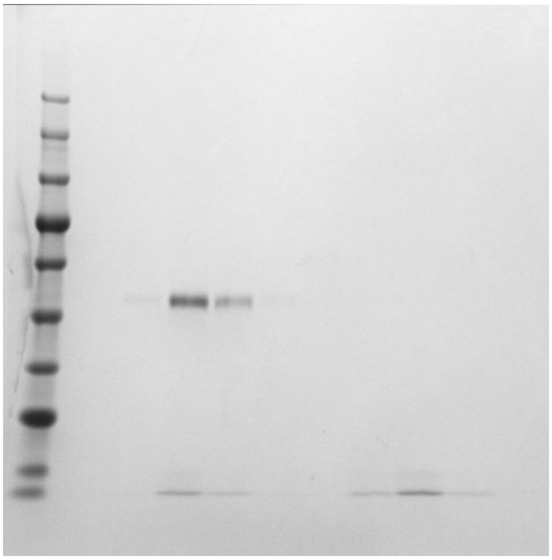

Supplementary Fig1. c
CBB staining

Supplementary Fig. 11 Uncropped gel/blot images from Figures.

Supplementary Table 1 Statistics of the data collection and refinement.

	S_8-meSRK-S_8-SP11	SeMet-S_8-meSRK-S_8-SP11
**Data collection**		
Space group	$P4_12_12$	$P4_12_12$
Cell dimensions		
a, *b*, *c* (Å)	143.56, 143.56, 194.40	142.69, 142.69, 194.76
α, β, γ (°)	90.0, 90.0, 90.0	90.0, 90.0, 90.0
Wavelength (Å)	0.9000	0.9791
Resolution (Å)	50.0-2.60 (2.64-2.60)*	50.0-3.50 (3.56-3.50)
No. of reflections		
Total	424,212	592,261
Unique	59,763	48,219
R_{merge}	0.10 (0.63)	0.14 (0.48)
$I/\sigma I$	41.3 (5.38)	18.2 (4.23)
Completeness (%)	94.9 (97.5)	100 (100)
Redundancy	7.1 (7.7)	12.3 (12.4)
CC1/2	†(0.87)	
**Refinement**		
Resolution (Å)	43.8-2.60 (2.69-2.60)	
R_{work}/R_{free}	0.220/0.251 (0.308/0.402)	
No. of atoms		
Protein	6,752	
Sugar	70	
Solvent	184	
Average B-factors (Å^2)		
Protein	61.7	
Sugar	87.2	
Solvent	50.6	
R.m.s deviations		
Bond lengths (Å)	0.012	
Bond angles (°)	1.17	
Ramachandran plot (%)		
Favored	94.0	
Allowed	6.0	
Outliers	0	

*Values in parentheses are highest resolution shell

†HKL2000 does not calculate total value of CC1/2

Supplementary references

1. Okamoto, S. *et al.* Self-compatibility in *Brassica napus* is caused by independent mutations in *S*-locus genes. *Plant J.* **50**, 391–400 (2007).
2. Hatakeyama, K., Takasaki, T., Watanabe, M. & Hinata, K. High sequence similarity between *SLG* and the receptor domain of *SRK* is not necessarily involved in higher dominance relationships in stigma in self-incompatible *Brassica rapa* L. *Sex. Plant Reprod.* **11**, 292–294 (1998).
3. Kusaba, M. & Nishio, T. Comparative analysis of *S* haplotypes with very similar *SLG* alleles in *Brassica rapa* and *Brassica oleracea*. *Plant J.* **17**, 83–91 (1999).
4. Takuno, S. *et al.* Effects of recombination on hitchhiking diversity in the Brassica self-incompatibility locus complex. *Genetics* **177**, 949–958 (2007).
5. Takuno, S., Oikawa, E., Kitashiba, H. & Nishio, T. Assessment of genetic diversity of accessions in Brassicaceae genetic resources by frequency distribution analysis of *S* haplotypes. *Theor. Appl. Genet.* **120**, 1129–1138 (2010).
6. Kusaba, M. *et al.* Self-incompatibility in the genus *Arabidopsis*: characterization of the *S* locus in the outcrossing *A. lyrata* and its autogamous relative *A. thaliana*. *Plant Cell* **13**, 627–643 (2001).
