# Supplementary material for: Mechanism of self/nonself-discrimination in Brassica self-incompatibility
Source: Nat Commun. 2020 Oct 1;11:4916. doi: 10.1038/s41467-020-18698-w (PMC7530648; doi:10.1038/s41467-020-18698-w)
Supplement: Supplementary file 3 — Descriptions of Additional Supplementary Files [file 41467_2020_18698_MOESM3_ESM.pdf]

Descriptions of Additional Supplementary Files

Supplementary Movie 1

Description: Time-lapse series of minimum intensity projections through a 3-D collagen gel with embedded fresh (left video) and cryopreserved (right video) expanded NK cells and K562 target cells. Magenta circles indicate exemplary non-motile NK cells, blue circles indicate exemplary K562 target cells.

Supplementary Movie 2

Description: Time-lapse series (total duration 30 min, recorded with a frame rate of 2/min) of maximum intensity projections through a 3-D collagen gel with embedded freshly expanded NK cells. The lower panel shows the output of the convolutional neural network used for automated cell tracking.

Supplementary Movie 3

Description: Time-lapse series (total duration 15 h) of minimum intensity projections through a 3- D collagen gel with embedded fresh expanded NK cells and K562 target cells. The left video shows an enlarged field-of-view around a K562 target cell that is attacked and killed by NK cells approx. 10 h after the beginning of the measurement. The right video shows the output of a convolutional neural network which is used as a prescreening tool to automatically find killing events. Note that each killing event and killing time point is manually verified for subsequent analysis.
