# Supplementary material for: Mechanism of self/nonself-discrimination in Brassica self-incompatibility
Source: Nat Commun. 2020 Oct 1;11:4916. doi: 10.1038/s41467-020-18698-w (PMC7530648; doi:10.1038/s41467-020-18698-w)
Supplement: Supplementary file 5 — Reporting Summary [file 41467_2020_18698_MOESM5_ESM.pdf]

nature research

Corresponding author(s): Yoshitaka Moriwaki, Toshio Hakoshima & Seiji Takayama

Last updated by author(s): Aug 26, 2020

Reporting Summary

Nature Research wishes to improve the reproducibility of the work that we publish. This form provides structure for consistency and transparency in reporting. For further information on Nature Research policies, seeAuthors & Referees and theEditorial Policy Checklist .

Statistics

For all statistical analyses, confirm that the following items are present in the figure legend, table legend, main text, or Methods section.

n/a	Confirmed	
☐	☒	The exact sample size (*n*) for each experimental group/condition, given as a discrete number and unit of measurement
☐	☒	A statement on whether measurements were taken from distinct samples or whether the same sample was measured repeatedly
☒	☐	The statistical test(s) used AND whether they are one- or two-sided<br>*Only common tests should be described solely by name; describe more complex techniques in the Methods section.*
☒	☐	A description of all covariates tested
☒	☐	A description of any assumptions or corrections, such as tests of normality and adjustment for multiple comparisons
☐	☒	A full description of the statistical parameters including central tendency (e.g. means) or other basic estimates (e.g. regression coefficient) AND variation (e.g. standard deviation) or associated estimates of uncertainty (e.g. confidence intervals)
☒	☐	For null hypothesis testing, the test statistic (e.g. *F*, *t*, *r*) with confidence intervals, effect sizes, degrees of freedom and *P* value noted<br>*Give P values as exact values whenever suitable.*
☒	☐	For Bayesian analysis, information on the choice of priors and Markov chain Monte Carlo settings
☒	☐	For hierarchical and complex designs, identification of the appropriate level for tests and full reporting of outcomes
☒	☐	Estimates of effect sizes (e.g. Cohen's *d*, Pearson's *r*), indicating how they were calculated

Our web collection on statistics for biologists contains articles on many of the points above.

Software and code

Policy information about availability of computer code

Data collection	UNICORN ver. 5.01 for gel-filtration analysis; ITC200 version 1.25.5 for ITC analysis; SPACE and BSS for data collection in SPring-8.
Data analysis	HKL2000 ver. 716.1, Phenix (including AutoSol, AutoBuild, Phaser, Phenix.refine and MolProbity) ver. 1.11.1-2575, PyMOL ver. 1.3 and 2.3, CCP4 (including areaimol) ver. 6.1.13, SHELX ver. 2014/2, Coot ver. 0.8.2 and ConSurf ver. 2016 for structure determination and analysis; ORIGIN ver. 7 for ITC analysis. ClustalW 2.1 for sequence alignment, PROMALS3D Web server for structure-based alignment (http://prodata.swmed.edu/promals3d/promals3d.php), Modeller 9.16 for homology modeling, Rosetta 3.9 for ab initio modeling, AMBER 18 for performing (accelerated) MD simulations, AmberTools 18 for MM-GBSA , MEGA X for generating phylogenetic trees.

For manuscripts utilizing custom algorithms or software that are central to the research but not yet described in published literature, software must be made available to editors/reviewers. We strongly encourage code deposition in a community repository (e.g. GitHub). See the Nature Research guidelines for submitting code & software for further information.

Data

Policy information about availability of data

All manuscripts must include a data availability statement. This statement should provide the following information, where applicable:

- Accession codes, unique identifiers, or web links for publicly available datasets
- A list of figures that have associated raw data
- A description of any restrictions on data availability

Structure factor and coordinate of S8-mSRK–S8-SP11 complex has been deposited in PDBJ (Protein Data Bank Japan) with the accession number 6KYW. There is no restriction on data availability.

Field-specific reporting

Please select the one below that is the best fit for your research. If you are not sure, read the appropriate sections before making your selection.

☒ Life sciences ☐ Behavioural & social sciences ☐ Ecological, evolutionary & environmental sciences

For a reference copy of the document with all sections, see nature.com/documents/nr-reporting-summary-flat.pdf

Life sciences study design

All studies must disclose on these points even when the disclosure is negative.

Sample size	Any statistical methods were used to predetermine the sample size. Sample size was determined by according to the customary practice. Best diffraction data set from a single crystal was used for the structure determination among the multiple data sets. In the pollination bioassay, more than ten samples were tested.
Data exclusions	In the pollination assay, all the data performed in the same day were discarded when the control compatible pollination was failed.
Replication	All biological and biochemical experiments were confirmed the reproducibility, except for the ITC and gel-filtration analysis because of the sample limitation.
Randomization	There is no experiment required for randomization.
Blinding	There is no experiment required for blinding.

Reporting for specific materials, systems and methods

We require information from authors about some types of materials, experimental systems and methods used in many studies. Here, indicate whether each material, system or method listed is relevant to your study. If you are not sure if a list item applies to your research, read the appropriate section before selecting a response.

Materials & experimental systems

n/a	Involved in the study	
☐	☒	Antibodies
☐	☒	Eukaryotic cell lines
☒	☐	Palaeontology
☒	☐	Animals and other organisms
☒	☐	Human research participants
☒	☐	Clinical data

Methods

n/a	Involved in the study	
☒	☐	ChIP-seq
☒	☐	Flow cytometry
☒	☐	MRI-based neuroimaging

Antibodies

Antibodies used	Anti-SRK antibody C1 was made in our lab and anti-rabbit IgG-HRP conjugate (BioRad, cat# 170-6515)
Validation	Anti-SRK antibody C1 was described in our previous paper (Shimosato et al., Plant Cell, 2007)

Eukaryotic cell lines

Policy information about cell lines

Cell line source(s)	Sf9 from Thermo Fisher Scientific.
Authentication	Nothing
Mycoplasma contamination	Not tested
Commonly misidentified lines (See ICLAC register)	Nothing
